# Activity of a novel, dual PI3-kinase/mTor inhibitor NVP-BEZ235 against primary human pancreatic cancers grown as orthotopic xenografts

**DOI:** 10.1038/sj.bjc.6604995

**Published:** 2009-03-24

**Authors:** P Cao, S-M Maira, C García-Echeverría, D W Hedley

**Affiliations:** 1 Division of Applied Molecular Oncology, Department of Medical Oncology and Hematology, Ontario Cancer Institute/Princess Margaret Hospital, University of Toronto, Ontario, Canada; 2 Novartis Institutes for Biomedical Research, Oncology Disease Area, Basel CH4002, Switzerland

**Keywords:** pancreatic cancer, primary xenograft, PI3K, drug treatment, mTor

## Abstract

The phosphatidylinositol-3-kinase (PI3K)/Akt signalling pathway is frequently deregulated in pancreatic cancers, and is believed to be an important determinant of their biological aggression and drug resistance. NVP-BEZ235 is a novel, dual class I PI3K/mammalian target of rapamycin (mTor) inhibitor undergoing phase I human clinical trials. To simulate clinical testing, the effects of NVP-BEZ235 were studied in five early passage primary pancreatic cancer xenografts, grown orthotopically. These tumours showed activated PKB/Akt, and increased levels of at least one of the receptor tyrosine kinases that are commonly activated in pancreatic cancers. Pharmacodynamic effects were measured following acute single doses, and anticancer effects were determined in separate groups following chronic drug exposure. Acute oral dosing with NVP-BEZ235 strongly suppressed the phosphorylation of PKB/Akt, followed by recovery over 24 h. There was also inhibition of Ser235/236 S6 ribosomal protein and Thr37/46 4E-BP1, consistent with the effects of NVP-BEZ235 as a dual PI3K/mTor inhibitor. Chronic dosing with 45 mg kg^−1^ of NVP-BEZ235 was well tolerated, and produced significant tumour growth inhibition in three models. These results predict that agents targeting the PI3K/Akt/mTor pathway might have anticancer activity in pancreatic cancer patients, and support the testing of combination studies involving chemotherapy or other molecular targeted agents.

Aberrant signalling by phosphatidylinositol-3-kinase (PI3K) is a prominent feature of pancreatic cancers, due to the high prevalence of abnormalities that regulate this pathway, including K-ras mutations that occur in approximately 90% of cases, increased expression of receptor tyrosine kinases like EGFR, and loss of PTEN (Korc, 1998; Ruggeri *et al*, 1998; Bardeesy and DePinho, 2002; Agbunag and Bar-Sagi, 2004; Asano *et al*, 2004; Chadha *et al*, 2006). The activation of downstream survival signalling pathways likely has an important role in the aggressive clinical features of pancreatic cancer, including resistance to chemotherapy. This is supported by recent results from a randomised clinical trial that showed a modest but significant improvement in patient survival when the EGFR inhibitor erlotinib was combined with the standard chemotherapy agent gemcitabine (Moore *et al*, 2007). Furthermore, we have previously shown that treatment with the natural product wortmannin resulted in decreased PKB/Akt phosphorylation in an orthotopic xenograft model of pancreas cancer, consistent with PI3K inhibition, and that combined treatment with wortmannin and gemcitabine resulted in greater acute induction of apoptosis, and improved tumour growth inhibition, compared to gemcitabine alone (Ng *et al*, 2001).

NVP-BEZ235 is a novel imidazoquinoline derivative that shows high selectivity for class I PI3K and the structurally related mammalian target of rapamycin (mTor; Maira *et al*, 2008). It has pharmacological properties that allow it to be tested for anticancer effects *in vivo*, and is well tolerated at dose schedules that result in decrease phosphorylated levels of the downstream target PKB/Akt. Furthermore, continuous oral dosing schedules with NVP-BEZ235 are growth inhibitory in human tumour xenograft mice and rat models (Maira *et al*, 2008).

In agreement with the experience from other groups, we have previously shown that primary xenografts can be established from the majority of pancreatectomy specimens and that when grown at the orthotopic site, they show typical histological features of pancreatic cancer (Ng *et al*, 2002; Rubio-Viqueira *et al*, 2006). These tumours therefore offer the opportunity for the near-clinical testing of novel molecular targeted agents in a controlled laboratory setting that allows detailed analysis of the relationships between the tumour characteristics, pharmacological effects, and anticancer activity. In the present paper we tested the effects of NVP-BEZ235 in a series of five early passage primary pancreatic cancer xenografts, and show that acute oral single doses suppress signalling targets downstream from PI3K/mTor in a time-dependent manner consistent with the pharmacokinetics of the compound, and that chronic treatment produces significant growth inhibition.

## Materials and methods

### Establishment of primary pancreatic cancer xenografts

Animal experiments were carried out using protocols approved by University Health Network Animal Welfare Committee. The establishment of the primary pancreatic cancer xenografts was carried out as previously described (Ng *et al*, 2002; Yau *et al*, 2005). Fresh pancreatic cancer samples that were superfluous to diagnostic needs were obtained from the University Health Network Tumour Tissue Bank according to institutional human ethical guidelines. Tumour fragments were initially implanted subcutaneously into the flanks of male SCID mice. Primary xenografts established from this material were subsequently transferred to the orthotopic site of 4- to 5-week-old mice by attaching tumour pieces from the previous passage to the surface of the exposed pancreas, by a small incision under general anaesthesia. Five primary pancreatic cancer xenografts, designated OCIP16, 17, 18, 19, and 21, were used at passage number 4–6 for these experiments.

### Characterisation of primary xenografts

Once sufficient tumour tissue was available, typically at passage number two or three, orthotopic tumours were rapidly excised and divided into roughly similar pieces. One of these was processed for protein extraction, one was snap frozen in liquid nitrogen, and one was fixed in formaldehyde followed by paraffin embedding. Cut tissue sections were stained with haematoxylin and eosin (H&E) for morphological assessment, and by immunohistochemistry for relevant protein markers including surface receptor tyrosine kinases and SMAD4 using appropriate primary antibodies. Codon 12/13 K-ras mutations were determined by gene sequencing. Activation of intracellular signalling proteins was assessed by immunohistochemistry using the following phosphospecific antibodies: Ser473 PKB/Akt; rabbit monoclonal obtained from Cell Signaling Technology (CST, Danvers, MA, USA); phosphorylated extracellular-regulated kinase (ERK) 1/2 (mouse monoclonal; CST). Signal transduction and activator of transcription (Stat) 3, Tyr705 (mouse monoclonal; CST) and Ser727 (rabbit polyclonal; CST). Tumour morphology was assessed by a pathologist, and the intensity of immunohistochemical staining for each of the tumour markers was scored from 0 (absent staining) to 3+ (strong staining).

### Drug treatment

**Figure 1:**
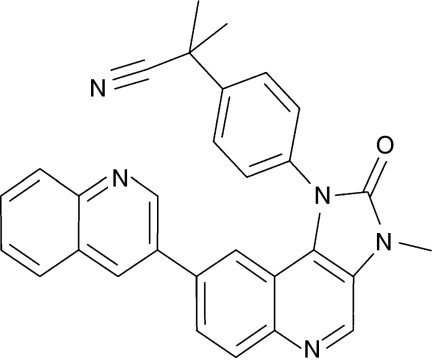
Structure of NVP-BEZ235.

NVP-BEZ235 (Figure 1) was obtained from the Oncology Department of the Novartis Institutes for Biomedical Research in Basel, Switzerland. Fresh stock solutions of the compound were prepared daily by dissolving in 1 volume of NMP (1-methyl-2-pyrrolidone; Sigma-Aldrich, Oakville, Ontario, Canada) in a 100°C water bath, then adding 9 volumes of PEG300 (Sigma-Aldrich) to give a final drug concentration of 12.5 mg ml^−1^.

To monitor the acute pharmacodynamic effects of NVP-BEZ235, five groups each of three randomly assigned tumour-bearing mice were treated with 50 mg kg^−1^ NVP-BEZ235 by oral gavage and killed at 0, 2, 4, 8, and 24 h. The tumours were rapidly excised and divided into pieces that were snap frozen in liquid nitrogen, or fixed in formaldehyde and then were paraffin-embedded, or processed for protein extraction.

To assess the anticancer effects, the NVP-BEZ235 dose was reduced to 45 mg kg^−1^, q.d., which is a dose and schedule that have been shown to be efficacious in *in vivo* mouse xenograft human cancer models (Maira *et al*, 2008). Two groups of 10–12 randomly assigned tumour-bearing mice were treated with NVP-BEZ235 or the vehicle control given by oral gavage daily for 5 days per week. Treatment commenced when the tumours were just palpable, and the duration was 3–5 weeks according the rates of tumour growth for the different models. The mice were monitored three times weekly by body weight and abdominal palpation, and for signs of distress, and killed according to animal welfare guidelines if they showed >10% loss of body weight or other signs of significant distress due to tumour growth or treatment toxicity. At the end of treatment the animals were killed and the tumours were harvested and weighed after removal of non-tumoural tissues. The tumours were then processed for further analysis, similar to the acute single-dose series.

### Measurement of NVP-BEZ235 effects

The pharmacodynamic effects of acute single doses were investigated by western blot and ELISA analyses of the tumour lysates. Minced tumour pieces were homogenised in 1 ml lysis buffer (50 mmol l^−1^ HEPES (pH 8.0), 10% glycerol, 1% Triton X-100, 150 mmol l^−1^ NaCl, 1 mmol l^−1^ EDTA, 1.5 mmol l^−1^ MgCl_2_, 100 mmol l^−1^ NaF, 10 mmol l^−1^ NaP_2_O_7_•H_2_O, 1 mmol l^−1^ NaVO_4_) containing protease inhibitor cocktail tablets (Roche Canada, Mississauga, Ontario, Canada) for 1 h on ice. Homogenates were clarified by centrifuging at 14 000 r.p.m. at 4°C for 15 min. Samples were then heated in sample buffer for 10 min at 95°C, run on 10% SDS–polyacrylamide gels, and transferred to PVDF membranes using the Mini Trans-Blot Electrophoresis Transfer Cell (Bio-Rad Laboratories, Mississauga, Ontario, Canada). Membrane blots were blocked for 1 h at room temperature with 10% BSA in TBS with 1% Tween 20 and then incubated overnight at 4°C with the following primary phosphospecific antibodies: Ser473 PKB/Akt (rabbit monoclonal from CST, 1 : 500 dilution), Ser21/9 GSK3 *α*/*β* (rabbit polyclonal from CST, 1 : 1000), Ser235/236 S6 ribosomal protein (CST; 1 : 7000) and Ser240/244 S6 ribosomal protein (rabbit polyclonal (CST; 1 : 1000), Thr37/46 4E-BP-1 (CST; 1 : 1000), Ser727 Stat3 (CST; 1 : 1000), and Tyr705 Stat3 (CST; 1 : 1000). The loading control was anti-actin (1 : 7000; Abcam, Cambridge, MA, USA). Following overnight incubation with the primary antibody, the blots were probed with either anti-rabbit polyclonal or anti-mouse monoclonal secondary antibodies labelled with horseradish peroxidase (GE Healthcare Biosciences Inc. Baie d’Urfe, Quebec, Canada) and then exposed to SuperSignal West Pico Chemiluminescent Substrate (Fisher Scientific, Ottawa, Ontario, Canada) according to the manufacturer's instructions.

To assess the effects of chronic drug administration on angiogenesis, proliferation, and apoptosis, paraffin-embedded sections of tumour tissues were stained by immunohistochemistry using antibodies to CD31, cyclin D1, p27, and cleaved caspase 3. The slides were then scanned using a ScanScope CS (Aperio Technologies Inc., Vista, CA, USA). Digital image analysis was carried out using the Aperio software, based on 10 fields of view of the tumoural area for each section, at × 10 magnification.

### Analytical procedure for quantification of BEZ235

Quantitative analysis of tumour samples for BEZ235 was performed with an HPLC/dual mass spectrometry (MS/MS) method. To each gram of tissue 1 ml of phosphate-buffered saline was added. The tissues were homogenised using an ULTRA-TURRAX (TP18-10; IKA, Staufen, Germany) homogeniser, keeping the material during the homogenisation as cold as possible by returning the homogenate to an ice bath between short (approximately 15 s) bursts. Proteins in tissue homogenate were precipitated by the addition of an equal volume of acetonitrile and processed further for chromatographic separation as described below. Stock solutions of the analyte BEZ235 (MW 469.6) and the structurally related internal standard (IS, MW 476.6) were prepared fresh daily at a concentration of 10 *μ*g ml^−1^ (analyte) and 1 *μ*g ml^−1^ (IS) in methanol/water (2 : 9, v/v). Further dilutions were prepared in the same solvent. Calibration samples were prepared in pooled homogenates of the corresponding tumours obtained from untreated animals. Tumour homogenate (25 *μ*l) was mixed with appropriate amounts of BEZ235 to deliver a nominal final concentration of 10–2000 ng ml^−1^. The quality control (QC) samples were spiked with the analytes to give a final concentration of nominally 40, 100, 400, and 1600 ng ml^−1^ respectively. A 50 *μ*l aliquot (1 *μ*g ml^−1^) of the IS was added to each calibration standard and to the QC samples and mixed on a vortex mixer for 15 s. After three times repeated protein precipitation by addition of an equal volume of acetonitrile followed by evaporation to dryness, the samples were re-dissolved in 100 *μ*l acetonitrile/water (1 : 9, v/v) containing 0.2% v/v formic acid. Sample injection volume was 5 *μ*l for the chromatographic separation.

The liquid chromatographic separations were carried out using an Agilent 1100 Series (Agilent, Palo Alto, CA, USA) HPLC system with vacuum degasser, capillary pump, and thermostated column compartment (40°C) combined with an automated injection device (CTC-PAL; CTC Analytics, Zwingen, Switzerland). As chromatographic separation matrix a RESECT Ultra Cyano reverse-phase HPLC column (column size 50 × 1 mm, particle size 3 *μ*m, preceded by a guard column: Phenomenex AJO-4304 phenylpropyl, size 4 × 2 mm) was used. Gradient mobile programming was used with a flow rate 60 *μ*l min^−1^; the mobile phase consisted of acetonitrile and 0.2% formic acid in water (95 : 5). The column eluent was directly introduced into the ion source of the triple quadrupole mass spectrometer Quattro Micro (Micromass, Manchester, UK) controlled by MassLynx 4.0 software. Electrospray positive ionisation (ESI(+)) multiple reaction monitoring was used for the MS/MS detection of BEZ235. After ESI(+) ionisation, the molecular–product transitions (*m/z* 470.35 → 443.25 product ion for BEZ235 and *m/z* 477.45 → 477.30 product ion for the IS) were monitored for the analyte and IS respectively. The calibration curve was prepared by adding the structurally related IS and appropriate amounts of analyte to mouse plasma or tumour tissue extract, covering a range from 2 to 2000 ng ml^−1^ with LOQ set to 10 ng ml^−1^ for plasma and 50 ng g^−1^ for the tumour tissue respectively (CV and overall bias less than 30%). Regression analysis was performed using QuanLynx 4.0 (Micromass) and Excel 2002 (Microsoft). Concentrations of unknown samples were calculated from the peak area ratio of the daughter ion of the analyte to the daughter ion of its IS (ordinate) against the nominal concentration (abscissa). Assay linearity was indicated by an overall regression coefficient of 0.9981.

### Statistics

All values are presented as mean±s.e. Comparisons between two groups (control *vs* NVP-BEZ235) were achieved using the two-tailed Student's *t*-test. The criterion for statistical significance was *P*<0.05.

## Results

### Characterisation of orthotopic primary pancreatic cancer xenografts

**Figure 2:**
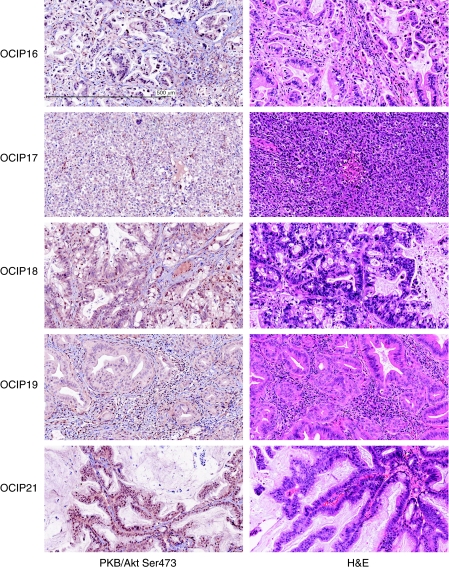
Histological sections of the orthotopically grown primary pancreatic cancer xenografts stained with haematoxylin and eosin (H&E; right panels), and by immunohistochemistry using anti-Ser473 Akt (left panels). Scale bar in the top left panel=500 *μ*m.

Histological examination of the H&E sections showed that the primary xenografts were adenocarcinomas with features similar to the original surgical specimens, with the exception of OIP17 that grew as sheets of poorly differentiated cancer cells. As seen in Figure 2, the histological features were more complex than those typically seen in xenografts established at the subcutaneous site from cell lines, as has been previously noted (Rubio-Viqueira *et al*, 2006). The tumours tended to be moderately well differentiated, with mucin production. They were organised into glandular structures, with a prominent fibrovascular stroma that in some cases comprised the bulk of the tumour. Cellular DNA content analysis by flow cytometry confirmed that in many of these tumours normal mouse cells accounted for >80% of the total cell population. By immunohistochemistry, phosphorylated Akt was readily detected in all five models (Figure 2). Staining for Ser473 Akt was also observed in the stroma of some of the xenografts, but this was less intense that seen in the tumour tissue. Immunohistochemical staining for the various growth factor receptors showed prominent surface membrane staining for EGFR in most cases, and variable expression of HER2 (ErbB2), c-Met (HGFR), and IGF-1R, often with marked intra-tumoural heterogeneity in staining intensity. The characteristics of the primary xenografts are summarised in Table 1.

### Acute single-dose effects of NVP-BEZ235

**Figure 3:**
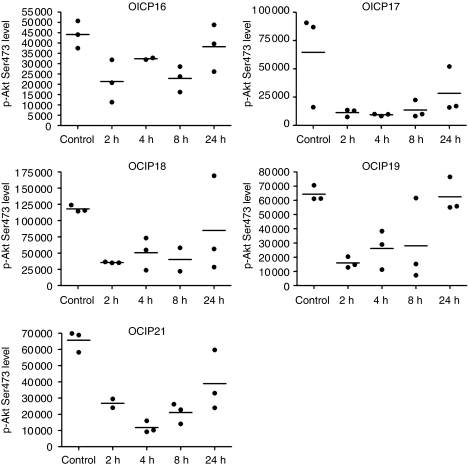
Effects of acute single doses of NVP-BEZ235 on phosphorylated Akt measured using an ELISA technique (10 *μ*g total protein per 50 *μ*l well). Groups of animals bearing the five primary xenografts at the orthotopic site were treated with 50 mg kg^−1^ NVP-BEZ235 and killed at the indicated time points.

**Figure 4:**
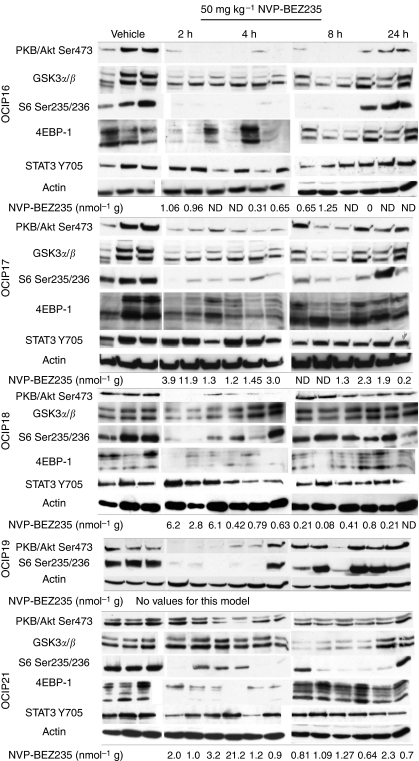
Western blots of tumour lysates obtained from triplicate primary xenografts at the indicated time points following an acute single dose of NVP-BEZ235, 50 mg kg^−1^, probed with primary antibodies to: Ser473 Akt, Ser21/9 GSK3*α*/*β*, Ser235/236 S6 ribosomal protein, Thr37/46 4EBP-1, Tyr705 Stat3, with actin loading controls. Tumour NVP-BEZ235 concentrations for each sample are indicated, with the exception of those of insufficient size for this (ND).

The acute single dose of 50 mg kg^−1^ NVP-BEZ235 administered by oral gavage was well tolerated. The levels of Ser473 phosphorylated PKB/Akt measured by ELISA showed considerable inter-tumoural heterogeneity, which reflects the complex nature of these tumours relative to subcutaneous implants of cell lines, but there was an obvious decrease in the mean values in all five models at 2 h, followed by recovery over 24 h (Figure 3). We also observed a decrease in Ser9/21GSK3*α*/*β* levels by western blot that followed a similar time course to p-Akt (Figure 4), consistent with inhibition of the PI3K/Akt pathway by NVP-BEZ235 in these models. We also found a time-dependent suppression of the downstream mTor targets Ser235/236 S6 ribosomal protein and Thr37/46 4E-BP1 in all five models, consistent with the action of NVP-BEZ235 as a dual PI3K and mTor inhibitor. Similar to the ELISA data shown in Figure 3, we observed considerable inter-tumoural heterogeneity within the triplicate samples, which to some extent tracked differences in the total protein levels, as shown in Supplementary Figure 1. Phosphorylated Stat3 was readily detected in all five models, consistent with previous reports showing aberrant activation in pancreatic cancer cells (DeArmond *et al*, 2003), but there was no significant decrease in the levels following treatment with NVP-BEZ235 (Figure 4), suggesting that the mechanisms of Stat3 activation are largely independent of PI3K signalling.

Tumour concentrations of NVP-BEZ235 were measured in all of the acute single-dose samples with the exception of OCIP19, where insufficient material was available due to their small size. Drug concentrations were found to be in average higher at 2 h than 24 h after administration (1.01 *vs* 0, 28.4 *vs* 0.50, 5.5 *vs* 1.44, 2.1 *vs* 1.20 nmol g^−1^, for OCIP16, 18, 17, and 21 respectively; Figure 4). These data show that different maximum concentrations are achieved in the four models but that in all cases, the compound is cleared form the tumour tissue with time, in agreement with the reported mouse pharmacokinetic profile (Maira *et al*, 2008).

### Tumour growth inhibition by NVP-BEZ235 treatment

**Figure 5:**
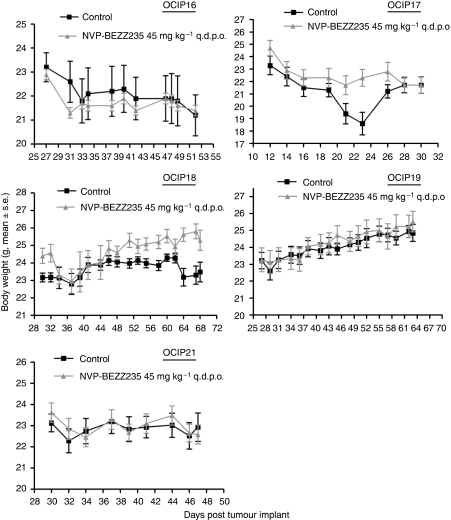
Animal weights during chronic administration of NVP-BEZ235 or vehicle control.

**Figure 6:**
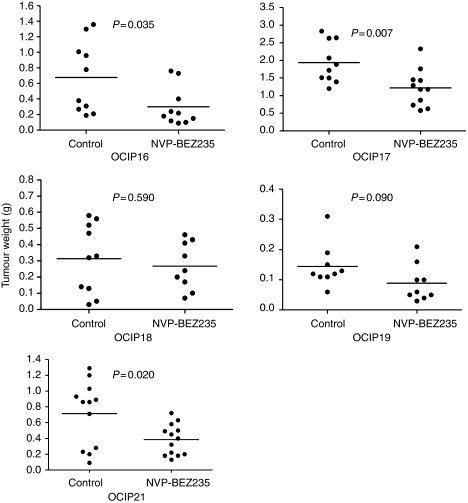
Tumour weights at the completion of chronic dose administration. Horizontal lines indicate the mean value for each group.

**Figure 7:**
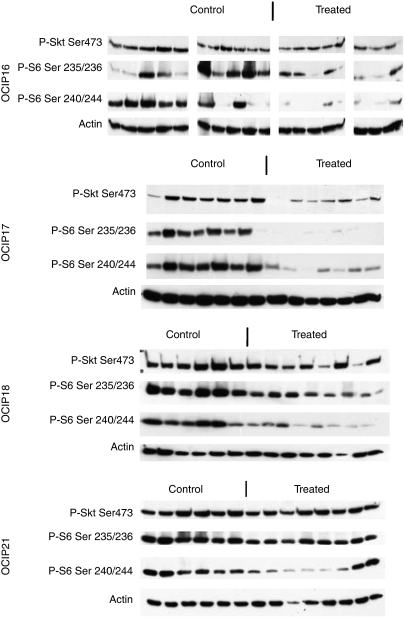
Western blots of tumour lysates obtained 2 h after the final dose of NVP-BEZ235 in the chronic dosing groups of animals, probed for Ser473 Akt and Ser235/236 and Ser240/244 S6 ribosomal protein, with actin loading controls. Owing to their small size, insufficient material was available for the OCIP19 tumours.

Daily oral administration of NVP-BEZ235 at a dose of 45 mg kg^−1^ was well tolerated in the tumour-bearing mice. Loss of weight occurred in the control groups of OCIP16 and 17, which was presumably the effect of the tumours, but the other groups of animals maintained their weight during the course of treatment and there were no statistically significant differences in the body weights of treated and control animals at the end of treatment in any of the five groups (Figure 5). Because of the location of the tumour in the orthotopic pancreas site, the tumour size was not measured until the end of the experiments when the animals were killed 2 h after the final dose. Consequently, at the end of the 3- to 5-week treatment, orthotopic tumours were dissected free of surrounding normal tissues and weighed. As shown in Figure 6, NVP-BEZ235 treatment of tumour models OCIP16, 17, and 21 resulted in statistically significant delay in tumour growth (56, 36, and 46% respectively) when compared with vehicle-treated controls. A fourth model, OCIP19, also showed growth inhibition, but this effect did not reach statistical significance, whereas there was no significant growth inhibition in OCIP18. By western blot, phosphorylated Akt and S6 were readily detected in these tumours excised 2 h after the final dose of NVP-BEZ235 (Figure 7), although it should be noted that the sample processing was considerably longer than that for the acute time course experiment shown in Figure 4, which might have affected these results. Compound concentrations in the tumour tissue 2 h after last treatment were in average of 1.11, 1.30, 0.81, and 1.16 nmol g^−1^, for OCIP16, 18, 17, and 21 respectively. Hence the lack of efficacy observed in the model OCIP18 cannot be accounted for by a lack of exposure. NVP-BEZ235 did not show accumulation over time in all the four tumour models tested, arguing that the efficacy is likely due to similar effects observed after a single-dose administration.

### Effects of NVP-BEZ235 on proliferation, apoptosis, and angiogenesis markers

**Figure 8:**
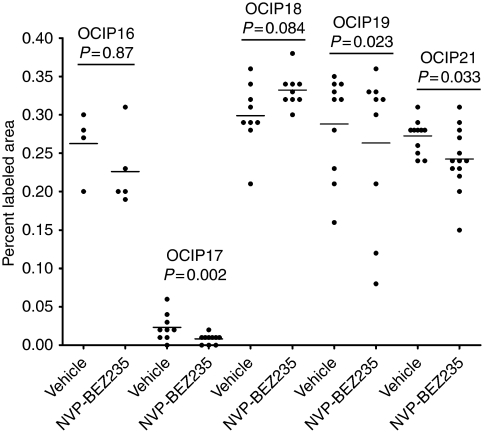
Cyclin D1 levels in tumour tissue following chronic dosing with NVP-BEZ235 or drug vehicle, measured using immunohistochemistry and semi-quantitative image analysis. Plots show the per cent labelled areas for individual tumours and the mean values, together with *P*-values based on unpaired two-tailed Student's *t*-tests.

To investigate the mechanisms of tumour growth inhibition by NVP-BEZ235, the tumours were labelled for cleaved caspase 3 and the proliferation markers cyclin D1 and p27 by immunohistochemistry, and the staining intensities were measured using semi-automated image analysis protocols. Cleaved caspase 3 showed low levels of staining relative to that previously observed by us in xenografts established from cell lines, and there was no significant increase in staining in any of the primary xenograft groups treated with NVP-BEZ235. There was a trend towards reduced labelling for cyclin D1 in the NVP-BEZ235-treated groups (Figure 8), and this was statistically significant in OCIP17, 19, and 21, whereas in the non-responsive OCIP18 model there was a non-significant increase in cyclin D1 in the NVP-BEZ235-treated animals. No significant effects on p27 were seen in any of the five primary xenograft models. Examination of newly formed blood vessels using CD31 staining showed that these were substantially present in the surrounding fibrovascular stroma, rather than invading into tumour masses. We tested several image analysis routines based on the number of CD31-stained blood vessels, their size, and distribution within tumour tissue and stroma. However, none of these yielded significant changes in patterns of blood vessel formation in any of the drug-treated tumours, relative to the vehicle controls.

## Discussion

The importance of aberrant PI3K signalling in cancer has been recognised for many years (Hennessy *et al*, 2005; Woodgett, 2005; Vogt *et al*, 2006; Workman *et al*, 2006). Apart from promoting cell growth, several signalling pathways diverge downstream from activated PKB/Akt that suppresses apoptosis. The enhancement of cell survival by PI3K signalling probably has a major function during early cancer development, and has also been linked to the high levels drug and radiation resistance seen in patients with pancreatic cancer (Ng *et al*, 2001; Bondar *et al*, 2002; Mackenzie, 2004; Brunner *et al*, 2005), although it should be noted that pancreatic cancers are heterogeneous, and the activation of additional signalling elements, including Stat3, NFκB, and hedgehog pathway, is also likely to be an important determinant of biological aggression (Bardeesy and DePinho, 2002; Arlt *et al*, 2003; DeArmond *et al*, 2003; Thayer *et al*, 2003). Experimental PI3K inhibitors can sensitise resistant cancer cells to cytotoxic agents or radiation, suggesting therapeutic potential in the clinic, but until recently *in vivo* testing was limited due to their toxicity or poor pharmacological properties. In contrast, the novel agent NVP-BEZ235 has suitable drug-like properties, and is currently been tested in phase I clinical trials in cancer patients. Given the high prevalence of genetic changes that can drive PI3K signalling in pancreatic cancers, we examined the pharmacodynamic and anticancer effects of NVP-BEZ235 in a series of five recently established primary pancreatic cancer xenografts.

Primary xenografts can be established in immune-deprived mice from the majority of pancreatic cancer resections. Particularly when grown at the orthotopic site, they show growth patterns including the formation of glandular structures embedded in a dense fibrovascular stroma that strikingly resemble those seen in surgical samples (Capella *et al*, 1999; Ng *et al*, 2002; Rubio-Viqueira *et al*, 2006). These tumours therefore appear to provide a realistic laboratory setting in which to test novel agents for treating a clinically intractable problem. In all models tested, acute single doses of NVP-BEZ235 decreased the phosphorylation of PKB/Akt, as well as its immediate downstream target GSK3, with maximum inhibition at the 2 h time point followed by recovery at 24 h, consistent with the pharmacokinetics of this compound (Maira *et al*, 2008). These findings confirm that NVP-BEZ235 is able to inhibit the activation of downstream PI3K effectors *in vivo*. In addition to its activity against class I PI3K, and like other PI3K inhibitors, such as wortmannin and LY294002 (Brunn *et al*, 1996), NVP-BEZ235 also blocks the catalytic activity of mTor. The kinase domain of this serine/threonine kinase has a high sequence similarity to PI3K. We observed decreases in the phosphorylation of downstream mTor targets, particularly the S6 ribosomal protein, that showed a similar time course to that seen with PKB/Akt phosphorylation. These findings suggest that mTor inhibition occurs *in vivo* at drug concentrations comparable to those that inhibit PI3K.

We tested for anticancer effects of NVP-BEZ235 using daily oral dosing, which is similar to treatment schedules commonly used in clinical trials testing novel molecular targeted agents. The primary end points for this part of the study were animal body and the tumour weight at the end of the treatment period. The tumour-bearing mice were also monitored by abdominal palpation, which gave a rough indication of tumour growth. In a pilot study we found that at a daily dose of 45 mg kg^−1^ NVP-BEZ235 was well tolerated by the SCID mice used for the xenografts, although weight loss occurred with more intensive treatment. This finding is consistent with previously reported *in vivo* efficacy studies from the Novartis group (Stauffer *et al*, 2007; Maira *et al*, 2008). Using the 45 mg kg^−1^ daily for 5 days per week dose schedule, we did not see increased toxicity relative to the drug vehicle control group during the treatment period. In three of the five primary xenografts, we observed statistically significant reduction in the tumour size or weight relative to the drug vehicle control group, which was consistent with the impression obtained from abdominal palpation that oral daily treatment with NVP-BEZ235 inhibited tumour growth. Non-significant growth inhibition was also seen in a fourth primary xenograft, OCIP19. This produced small, slow-growing tumours, and a statistically significant effect might have been lost due to measurement error. A fifth model, OCIP18, was insensitive to growth inhibition by NVP-BEZ235. Because the acute effects on PKB/Akt phosphorylation were comparable to those seen with the other primary xenografts, the lack of response to NVP-BEZ235 treatment is likely explained by the activity of additional oncogenic signalling pathways in OCIP18, rather than failure to inhibit the drug target. Perhaps relevant to this, the levels of cyclin D1, which can be regulated by several mechanisms downstream of PI3K/mTor, showed a decrease in all models with the exception of OCIP18 (Figure 8).

It is likely that the use of orthotopically grown early passage primary xenografts provides a better prediction of clinical activity, relative to *in vivo* sensitivity testing based on pancreatic cancer cell lines. Overall, the effects of NVP-BEZ235 appear less dramatic in these models relative to those seen in xenograft established from cell lines (Maira *et al*, 2008; Serra *et al*, 2008), which are typically poorly differentiated and rapidly proliferating. We also failed to show significant anti-angiogenic effects in the orthotopic primary pancreatic cancer xenografts, which is in contrast with other groups who have reported pronounced anti-angiogenic effects by mTor inhibitors used to treat xenografts established from pancreatic cancer cell lines (Azzariti *et al*, 2008; Manegold *et al*, 2008). Again, we feel that this might reflect differences in the growth patterns seen using the orthotopic primary xenografts. Interestingly, Schnell *et al* (2008) have recently shown that NVP-BEZ235 inhibited microvessel permeability in a syngeneic rat mammary carcinoma model, but we did not test for this effect in the present study. Nevertheless, the response rate of 3/5 seen in the present study appears greater than that recently reported for other molecular targeted agents in primary pancreatic cancer xenografts (Rubio-Viqueira *et al*, 2006), suggesting that dual PI3K/mTor inhibitors like NVP-BEZ235 might have useful anticancer effects in pancreatic cancer patients.

It remains to be determined if the daily oral dosing schedule for NVP-BEZ235 used in this study will be optimal in the clinic. Results from *in vitro* experiments suggest that the anticancer effects of PI3K inhibitors are dependant on the extent and duration of target inhibition. However, the acute single-dose experiments showed that the NVP-BEZ235 dose used did not completely inhibit PKB/Akt in tumour tissue, and the effect was of relatively short duration. Furthermore, phosphorylated PKB/Akt was detectable in the tumour samples obtained 2 h after the final dose in the chronic treatment group, indicating incomplete drug target inhibition. It is unlikely that complete target inhibition could be sustained over long periods in animal models or cancer patients, because PI3K signalling is critical in the maintenance of normal tissues. However, it is possible that higher doses given intermittently might give a better therapeutic ratio by producing more complete PI3K inhibition in the tumour, while allowing time for normal tissue recovery.

An alternative strategy would be to combine a PI3K inhibitor like NVP-BEZ235 with other anticancer agents. For example, we have previously shown that this can markedly enhance sensitivity to the standard agent gemcitabine when used in an intermittent dose schedule (Ng *et al*, 2001). However, in the longer term it is likely that synergistic interactions could be obtained combining PI3K inhibitors with agents targeting other signalling pathways that are aberrantly regulated in pancreatic cancers. The clinical development of treatment protocols incorporating multiple molecular targeted agents is problematic, because the optimum combination would depend on the specific pattern abnormalities in a particular cancer patient. Hence we believe that the long-term strategy needed for an intractable problem such as pancreatic cancer requires closer integration between basic science and clinical oncology, including the preclinical testing based on models similar to those used in the present study.

## Figures and Tables

**Table 1:** Characterisation of the primary pancreatic cancer xenografts

**Tumor ID**	**Histological type**	**EGFR**	**Her2**	**HGFR**	**IGF-1R**	**p-Akt**	**SMAD4**	**K-ras**
OCIP16	Adenocarcinoma	++	++	+	++	++	+++	Codon 12 mutation
OCIP17	Poorly differentiated	−	−	−	++	+	+++	w/t
OCIP18	Adenocarcinoma	+++	++	++	++	++	−	w/t
OCIP19	Adenocarcinoma	++	+	+	+	+	−	Codon 12 mutation
OCIP21	Adenocarcinoma	+++	+++	++	+++	+++	−	w/t

w/t=wild type.
