# Supplementary figures and images for: Activity of a novel, dual PI3-kinase/mTor inhibitor NVP-BEZ235 against primary human pancreatic cancers grown as orthotopic xenografts
Source: Br J Cancer. 2009 Mar 24;100(8):1267–76. doi: 10.1038/sj.bjc.6604995 (PMC2676548; doi:10.1038/sj.bjc.6604995)

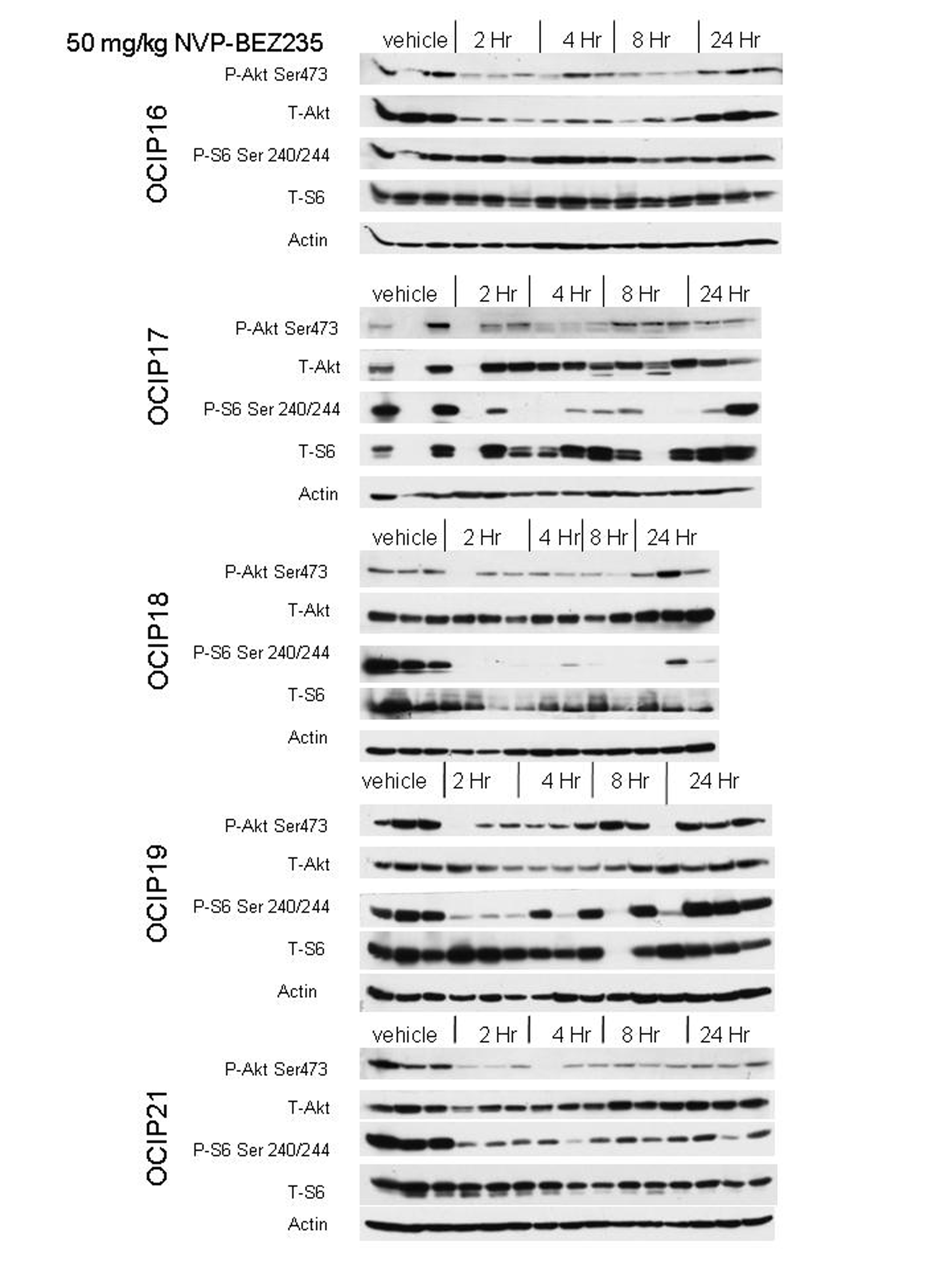

Supplement: Supplementary Figure 1 [file 6604995x1.tif]
