# Supplementary material for: Activity of a novel, dual PI3-kinase/mTor inhibitor NVP-BEZ235 against primary human pancreatic cancers grown as orthotopic xenografts
Source: Br J Cancer. 2009 Mar 24;100(8):1267–76. doi: 10.1038/sj.bjc.6604995 (PMC2676548; doi:10.1038/sj.bjc.6604995)
Supplement: Supplementary Figure Legend [file 6604995x2.doc]

**Figure Legend**

**Supplementary Figure 1**

Western blots of tumour lysates obtained from triplicate primary xenografts at the indicated time points following an acute single dose of NVP-BEZ235, 50 mg/kg, probed with primary antibodies to: Ser473 Akt, total Akt, Ser 240/244 S6 ribosomal protein, total S6 ribosomal protein, with actin loading control. Individual tumours and sequence are the same as in Figure 4, but the lysates were obtained from frozen tissue blocks from a different piece of the tumour.
